# An exploration of markers of microvascular dysfunction in kidney transplant recipients randomized to belatacept: no clinical impact of CNIs on endothelial function

**DOI:** 10.3389/frtra.2026.1812847

**Published:** 2026-06-11

**Authors:** Edwin O. W. Bredewold, Rudmer J. Postma, Joe Chan, My H. S. Svensson, Annette Bruchfeld, Johannes W. de Fijter, Hans Furuland, Anders Hartmann, Lars Mjornstedt, Karin Skov, Inga soveri, Soeren S. Sorensen, Bengt Fellstrom, Jacques M. Duijs, Roel Bijkerk, Joris I. Rotmans, Anton-Jan van Zonneveld

**Affiliations:** 1 Department of Transplantation and Nephrology, Leiden University Medical Center (LUMC), Leiden, Netherlands; 2 Department of Nephrology, Akerhus University Hospital, Lorenskog, Norway; 3 Institute of Clinical Medicine, Akerhus University Hospital, Lorenskog, Norway; 4 Department of Clinical Medicine, Aalborg University Hospital, Aalborg, Denmark; 5 Department of Health, Medicine and Caring Sciences, Linkoping University, Linkoping, Sweden; 6 Department of Clinical Science, Intervention and Technology, Karolinska Institutet, Stockholm, Sweden; 7 Department of Medical Sciences, Uppsala Universitet, Uppsala, Sweden; 8 Department of Transplantation Medicine, University of Oslo, Oslo, Norway; 9 Department of Surgery, Goteborg University, Goteborg, Sweden; 10 Department of Renal Medicine, Aarhus University Hospital, Aarhus, Denmark; 11 Department of Clinical Medicine, Rigshospitalet, Copenhagen University, Copenhagen, Denmark

**Keywords:** belatacept conversion, calcineurin inhibihibitor nephropathy, endothel dysfunction, kidney tranplantation, randomiced clinical trial

## Abstract

**Introduction:**

Side effects of calcineurin inhibitors (CNI) comprise hypertension, diabetes and nephrotoxicity, and endothelial dysfunction (ED) has often been implicated. Belatacept, a co-stimulation blocker, may offer a more favourable vascular profile.

**Methods:**

We investigated markers of endothelial function in blood samples from kidney transplant recipients (KTR) that were converted from CNI to belatacept. We assessed established markers of ED including soluble thrombomodulin, P-selectin and angiopoietin 1 and 2. In addition, we assessed expression levels of microRNAs (miRNAs), which have been identified as discriminative markers for ED, rejection and renal fibrosis in the setting of transplantation. Lastly, we used patient samples and CNI-dilution series in a validated model of ED in which changes in morphological characteristics of endothelial cells were evaluated.

**Results:**

Unexpectedly, we found no differences in our assays between both groups. We did observe dose-related effects of CNI on endothelial cell (EC) morphology upon direct exposure of EC to tacrolimus and ciclosporin A, but a similar effect was not seen when plasma samples of KTR who were converted to belatacept were compared with samples from KTR on CNI's.

**Discussion:**

These data suggest that CNI-side-effects bundled under the term “CNI-toxicity” are not derived from a significant clinical effect of CNI on endothelial function.

## Introduction

Calcineurin inhibitors (CNI) are used in solid organ transplantation since the nineteen-seventies, and have contributed to significantly lower rates of rejection, and longer graft survival (1). Guideline committees have implemented CNI in combination-regimens, together with mycophenolic acid (MMF or a mammalian target of rapamune inhibitor (mTORi), and steroids (2). Notoriously, CNIs can affect GFR, as well as a number of long-term risk factors for kidney function decline such as diabetes, dyslipidemia and hypertension (3–5). In fact, the term “CNI-toxicity” refers to a clinical picture that includes decreased GFR, hypertension and sometimes hyperkalemia and liver enzyme-disturbances, occurring after introduction or dose-increment of CNIs. Chronic use can be manifested by irreversible loss of GFR and graft diysfunction. Histologically, its use is associated with arterial hyalinosis, interstitial fibrosis and tubular atrophy (6).

An alternative to CNI-based regimens is available in the form of belatacept, which is a co-stimulation blocker, designed by coupling the extracellular part of cytotoxic T-lymphocyte antigen 4 (CTLA4) to the Fc-chain of IgG. In the past fifteen years, various clinical studies with belatacept have shown that it carries a relatively high risk of rejection in the first year after transplantation, but a clear benefit for renal function, with glomerular filtration rate (GFR) consistently higher when compared to patients on CNIs in conversion-trials (7–9). Because of this, most centers that use belatacept introduce it later after transplantation, or only prescribe it to patients that have severe CNI-related toxicity (10, 11).

Not much is known about the mechanisms through which CNIs exert toxicity. Endothelial dysfunction (ED) is often implied, based on data from preclinical studies. Specifically, CsA induces acute efferent arteriolar vasoconstriction due to disturbance of the balancing effects of endothelium-relaxing factor (NOS) and prostaglandin E2 on the vasodilating side and endothelin-1 (ET-1) and thromboxane on the other (12–14). Similar effects on ET-1 production and release by CsA have been described for TAC (15). The finding of lower blood pressures and reduced arterial stiffness are clinical indications of ED being favorably influenced by conversion to belatacept (16–19). Wang et al. have shown an improvement of several measures of ED and renal hemodynamics in a rat model of diabetic nephropathy, indicating a direct effect of CTLA-Ig on renal parenchyma (20). Besides a direct effect it is assumed that part of any conversion effect is due to discontinuation of CNI (21).

At the Leiden University Medical Center (LUMC), we have participated in two randomized controlled trials (RCT) exploring the effect on cardiovascular risk factors and renal function after conversion from CNI to belatacept (9, 16). Both studies had similar in- and exclusion criteria and follow up, providing a chance to investigate features of ED in kidney transplant recipients (KTR) converted to belatacept and weaned off CNI. We hypothesized that patients converted to belatacept would exhibit changes indicating an improvement in ED associated with CNI, and we expected those changes to be early reflections of the clinical benefit that belatacept-treated patients have shown in previous trials. Therefore, we used blood samples of patients in either of the two conversion trials, and compared samples before conversion and one year after. With these, we performed a broad investigation of markers of ED, ranging from established biomarkers as angiotensin I and II (Ang-1/Ang-2), soluble thrombomodulin (s-TM) and P-selectin (P-sel), to an exploratory investigation of microRNAs (miRNAs) previously associated with ED, renal fibrosis and restoration of kidney function after kidney transplantation. Furthermore, we explored the effects of different concentrations of calcineurin inhibitors on cultured endothelial cells, as well as the effects of plasma samples from participants on an innovative endothelial cell morphological profiling assay. This assay was previously validated in our hospital in a cohort of patients with cirrhosis, and in other conditions associated with microvascular disease (22). This study could provide insight into the reversibility of early ED, and contribute to our knowledge of mechanistic differences between belatacept and CNI.

## Methods

Plasma samples of KTR's of both RCT's [EudraCT no. 2013-001178-20 (SMR-2729) and NCT01820572 (IM103-116)] stored at the Leiden University Medical Centre were used for this study.. Both studies involved KTR's that were randomized to convert from standard immune suppression with CNI (93% had TAC, 7% CsA) to belatacept, or to continue with CNI. The inclusion- and exclusion criteria, follow-up and background immunosuppression were very similar in both studies (9, 16). The standard regimens in all participating centers consisted of prednisone (5 mg standard-dose) and MMF. Blood samples were collected immediately before and at 12 months after randomization, and stored at - 80° Celsius. Informed consent was provided upon participation in the respective trials.

### Measurement of biomarkers of vascular function

Plasma was isolated from 5 mL of patient-derived venous blood after being freshly drawn into EDTA-coated vacutainers and mixed carefully head-to-head to ensure proper exposure of the blood to the EDTA. Subsequently, samples were centrifuged at room temperature at 150 g for 15 min. Platelet-rich plasma (PRP) was isolated and centrifuged again at 2000g for 10 min. Aliquots of 500 μL plasma were transferred into freezer tubes and stored at −80˚C. Serum was collected by allowing full blood to coagulate for 30 min. The supernatant was then centrifuged at 4 ˚C at 2000g for 10 min. After that, samples were stored in aliquots at −80˚C.

For this study, plasma was defrosted and levels of Ang-2, P-sel and s-TM were determined using Enzyme-Linked Immunosorbent Assay (ELISA) according to manufacturers' protocol (R&D Systems, Minneapolis, MN, USA; catalogue number (cat#) DANG20, DPSE00, DTHBD0). For measurement of Ang-1, serum was used (R&D Systems, Minneapolis, MN, USA; cat# DANG10).

### Extraction, profiling and measurement of miRNAs

A total of 200 μL of plasma was mixed with 800 μL of Trizol LS reagent (Invitrogen, Breda, The Netherlands, cat# 10296028). Total RNA was then isolated using the RNeasy Micro Kit (Qiagen, Venlo, The Netherlands, cat# 74004) with a modified protocol. Chloroform was added to the plasma/Trizol LS mixture, followed by centrifugation for 15 min at 12000rpm. For extraction of RNA, further centrifugation and washing steps were performed.

After extraction, reverse transcription of total RNA was performed using the micro-RNA Reverse Transcription Kit (TaqMan, Applied Biosystems, Foster City, CA, USA, cat# 4366596) in combination woth Megaplex RT Primers Pool A v2.1 (Cat# 4399966). Subsequently, cDNA was pre-amplified using Megaplex PreAmp primer pools A V2.1 (Applied Biosystems, cat# 4399233) according to the manufacturer's instructions. The resulting DNA was then loaded onto custom-made Megaplex cards to determine the expression profile of a selected set of miRNAs in serum, with RNU44, RNU48 and U6 serving as controls. These Megaplex arrays were run and analyzed on a 7900HT Fast Real-Time PCR System (Applied Biosystems).

Ct values for designated miRNAs were normalized to the median expression of miRNA of each megaplex-plate. Expression of individual miRNA-levels was presented as fold changes compared to one index case at baseline, using the standard *ΔΔ*Ct method. The methods for extraction, profiling and measurement of miRNAs were similar to the protocol used by Bijkerk et al. (23).

### Selection of miRNAs

Table 1 gives an overview of the selected miRNAs, as well as the biological function with which it is implicated, as well as a literature reference if available. We chose miRNAs associated with AD, allograft dysfunction or CNI-toxicity, with a preference for those we found to be discriminative for diabetes and restoration of renal function after transplantation. For capacity reasons, we selected 20 miRNAs.

**Table 1:** Selected miRNAs.

MiRNA	Biological process	Reference
miR-92a, miR-210, miR-30c, miR-27a, miR-126, miR-130b, miR-132, miR-200c, miR-320, miR-340, miR-660	Endothelial dysfunction, renal fibrosis, restoration of renal function.	(24–30)
miR-199a-3p, miR-214, miR-21, miR-150, miR-155, miR-192, miR-200b, miR-142-5p	CNI-toxicity, allograft dysfunction	(31–34)
miR-16	Renal fibrosis	(35)

### Endothelial cell morphology assay

The endothelial cell morphological profiling assay has been described previously (22, 36). In short, human umbilical vein endothelial cells (HUVEC) were obtained and cultured as described previously (22). Low-passage HUVECs were thawed and cultured in endothelial cell growth medium 2 (EGM-2) (PromoCell C-22111) supplemented with 1% Penicillin/Streptomycin (Gibco 15070-063) in a gelatin (1% in PBS, Merck; 104078) coated T75 flask for 3 days. Cells (20,000/well) were then seeded in a gelatin coated 96-wells plate (PerkinElmer PhenoPlate, 6055300) 4 days prior to the experiment.

Two experiments were performed using this assay; 1: CNI titration series, and 2: patient-derived plasma exposure.

Morphological responses to different concentrations of CNI were investigated for CsA (Neoral®, Novartis Pharma BV, Switzerland) and TAC (Prograft®, Astellas Pharma BV, Japan). A titration series of 0 (zero), 5, 10, 20, 40, 80, 160, 320, 640, 1280, 2560 and 5120 mcg/L (CsA) and 0, 0.5, 1, 2, 4, 8, 16, 32, 64, 128, 256 and 512 ng/mL (TAC) was performed in endothelial cell basal medium (PromoCell C-22111), supplemented with 5% healthy human serum. Both dilution series are compatible with blood-concentrations that represent subclinical, clinical, and toxic levels. The cultured endothelial cells were incubated with the different mixes for 18 h, followed by immunofluorescent staining of the cells.

Morphological responses to patient-derived plasma were investigated by exposing cultured endothelial cells to plasma collected from patients treated in the LUMC in either of the two randomized trials. K_2_EDTA plasma was stabilized and recalcified by adding 0.5 µM recombinant Hirudin (ABCAM ab201396), 25 µg/mL Corn trypsin inhibitor (HTI CTI-01), and 1.85 mM calcium-dichloride (CaCl2) (Merck 1.02381) to a K_2_EDTA plasma volume equaling 25% of the total volume. Endothelial cell basal medium (EBM) (PromoCell C-22211) containing 1:100 insulin transferrin selenium supplement (ITS) (Gibco 41400) was added to obtain the final volume. The cultured endothelial cells were incubated with the different plasma mixes for 18 h, followed by immunofluorescent staining of the cells.

Cells were stained according to the previously published protocol, modified to include a staining for von Willebrand factor (vWF). Cells were incubated with MitoTracker Deep Red FM (InVitrogen M22426) 1:2000 for 30 min, followed by incubation with 4% paraformaldehyde (Alfa Aesar J61899) for 10 min, and 30 min incubation with blocking buffer [2% (w/v) BSA+0.5% Glycine (Merck 1.04201) + 1% (v/v) Triton X-100 in PBS]. Afterwards, wells were incubated with primary antibodies against VE-Cadherin (BD 555661, 2 µg/mL) and vWF (DAKO a0082, 1:1000) in PBS with 2% BSA for one hour, followed by incubation with 488 Alexa-fluorophore labeled Donkey anti-mouse antibody (Invitrogen A-11001, 2 µg/mL), Qdot 625 labeled Goat anti-rabbit antibody (Invitrogen A-10194, 1:500), 1:500 Rhodamine Phalloidin (Invitrogen R415), and 1:1000 HOECHST 33258 (Molecular Probes) in 50 mM Borate Buffer, pH = 8.3 with 2% BSA+0.05% glycine for one hour. Wells were washed with borate buffer, and stored under borate buffer for imaging. Max-projections of 9 z-steps with 0.7 µm step size were acquired using a high content confocal microscope (Molecular Devices, ImageXpress™ Micro Confocal) at 20× magnification (Nikon Plan Apo Lambda; NA = 0.75), using a 60 µm pinhole. Six sites without overlap were imaged per well.

For each image, a focus-score metric, intensity sum, median, standard deviation, and the 0.01, 0.25, 0.75, 0.99 quantiles were computed. Results were plotted in histograms comprised of all images for a given channel to assess outliers. Visual inspection of outliers was performed to assess image quality before discarding. Images that contained artifacts (e.g., out-of-focus images, debris, clipping/saturation artifacts) were discarded. Sites that contained failed images in a single channel were discarded altogether. Following image quality control, images were corrected for uneven illumination and vignetting. For a description of the IF staining, imaging, image control, we refer to a previous publication (22).

### Image analysis

Single-cell morphological profiles were extracted from the images using R (version 4.1.1) (37), the R-package *EBImage* (version 4.29.2) (38), and in-house scripts. In short, individual nuclei were identified from Dapi-channel images and used as seeds to identify the cell borders using the VE-Cadherin signal. To filter out debris, upper and lower thresholds were applied for the nuclei size. R-package EBImage “compute” functions were used to compute morphological features. Functions to extract distribution and colocalization were based on the procedure by Verbeek et al. (39).

### Data analysis

Data analysis concerning patient data was performed using R(V 4.1.1.) Qualitative data are described using frequencies and percentages. Quantitative variables are described using mean (standard deviation) or median (IQR) when appropriate. Comparisons between treatment groups were performed using ANOVA and student's t-test for continuous variables. A *p*-value < 0,05 was considered to be statistically significant. R-packages *haven* (V2.5.4), *ggplot2* (V3.5.1) and *factoextra* (V1.0.7) were used to perform principal component analysis (PCA) of miRNAs.

Data analysis of the morphological data was performed in Python version 3.7.6, using functions from the Scikit-learn package (40). The approach was as follows: Z-score normalization was applied to the single-cell data of each plate dataset separately (36). Afterwards, datasets were combined. Single-cell profiles were reduced in dimension by factor analysis (41), capturing about 80% of total variance, resulting in 18 factors. Mean profiles were created by averaging the cells for each patient/time point in the reduced subset. Resulting profiles were scaled to 0-mean and unit variance. These profiles were used for modelling the endothelial morphological response, either by principle component analysis, linear discriminant analysis, or 5-fold cross-validated support vector machine (SVM) with rbf-kernel.

### Statistics for vascular markers and miRNAs

For analysis of markers of vascular function (concentrations in ng/mL, continuous variables), we used Graphpad prism 10.2.3. We used a mixed-effects model for longitudinal analysis (baseline vs. T12). Fisher's LSD (least significant difference) was used for *post hoc* analysis. For between-group comparisons we performed unpaired t-tests. A *p*-value of < 0,05 was considered statistically significant.

For miRNAs, we calculated fold changes (FC) for each selected miRNA. We compared average FC per miRNA between baseline and T12 using unpaired two-sample t-tests when data were normally distributed. If not we performed Wilcoxon rank-sum-test. *P*-values were corrected for multiple testing using the FDR-method. We made volcano plots to visualize effect size and significance. We performed principal component analysis to explore clustering patterns within miRNA-profiles. All analyses were done using R version 4.3.2 (*ggplot2*, *enhancedvolcano*-packages).

## Results

### Data collection and patient characteristics

Samples of 100 patients from 4 different countries were used. From the Netherlands, there were 29 patients from SMR-2729, and 10 from IM-103-116 (9, 16). All other patients were recruited from SMR-2729 (20 from Denmark, 14 from Norway and 27 from Sweden). Patients were eligible for both studies after 3 and 6 months respectively. Median time after transplantation at recruitment was 26 months. Patients in both studies were either randomized to convert to belatacept, at a dose of 5 mg/kg at day 1, 15, 29, 43, 57 and every 28th day thereafter, or to continue on CNI according to their local protocol. Data on traditional vascular markers Ang 1/2, STM and P-selectin were obtained from all patients, at baseline (T0) and 12 months later (T12). The measurements were performed shortly after completion of the study in 2019. To perform the miRNA-measurements, we used samples that had been stored for more than six years, and defrosted only after definite resolution of the COVID-19 pandemic. Unfortunately, at that time we found that some samples had lost their identification label. Therefore, we used only those samples of which the labels were readable, and of which one was available at baseline (before randomization) and one year after randomization (T12). After normalization of the miRNA-data, only samples with a cycle threshold of lower than 35 were used. In total, samples of 70 patients with measurements from at least two time-points were included.

For the cell-painting assays, we used only patient-samples from local (Leiden) patients that had participated in the RCT's. From that group, we possessed samples collected at baseline, at 6 months after randomization and after one year (T0, T6, T12). Data from all 100 patients are depicted in Table 2. Data from the smaller group of 70 (miRNA) and 39 (cell-painting) patients are available as Supplementary Tables S1 and S2. Of note, the patients in the control group of all cohorts had slightly better GFR despite randomization, whereas all other clinical characteristics were evenly distributed. 93.1% of patients used TAC before randomization (89.6% in control group, 96.3% in belatacept).

**Table 2:** Baseline-characteristics for all patients.

Variable	CNI (48)	Belatacept (52)
Age (mean, SEM)	57 (1.9)	57 (2.0)
Female sex (n,%)	11 (22%)	16 (29%)
LDL (mean, mmol/L, SEM)	2.7 (0,1)	2.7 (0,1)
Months since transplantation	26.6 (3)	27.7 (2)
Dialysis vintage in months	15.6 (3)	15.6 (2)
History of smoking	30 (60%)	24 (45%)
Diabetes	8 (16%)	13 (23%)
Systolic BP (mmHg)	134	136
Diastolic BP (mmHg)	82	83
eGFR (mL/min/1.73m2)	61 (2.8)	52 (2.0)
CsA before randomization	5 (10.4%)	2 (3.8%)

No significant change in traditional biomarkers of vascular health after conversion from tacrolimus to belatacept.

**Figure 1:**
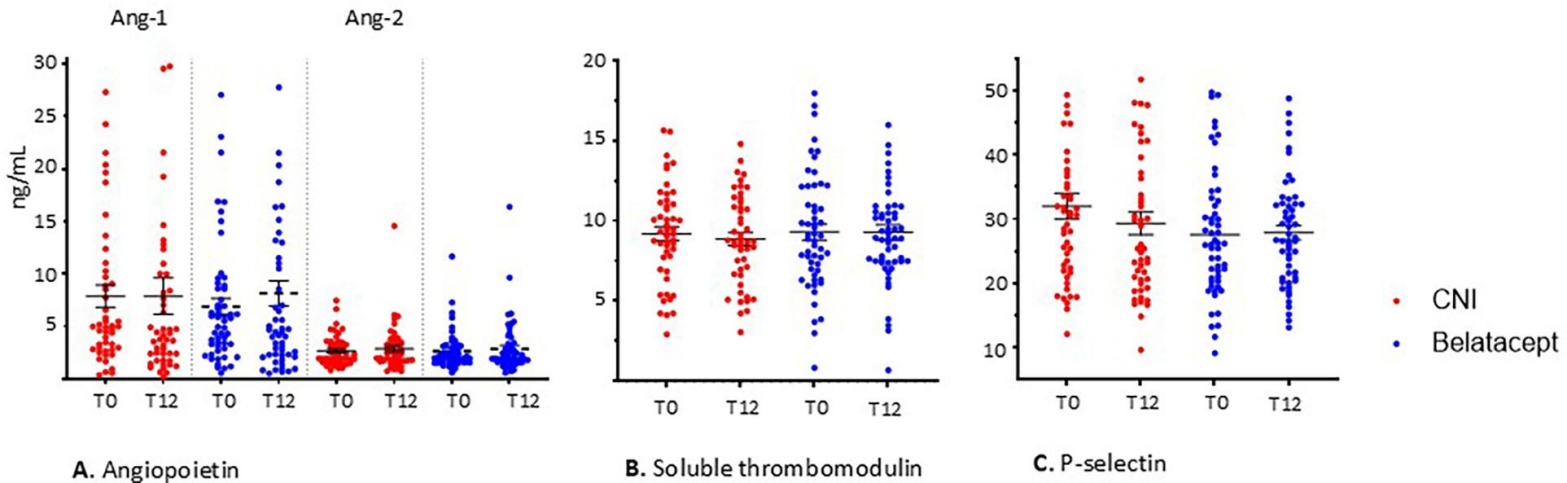
**(a)** levels of angiopoietin l and 2. **(b)** Levels of soluble thrombomodu lin. **(c)** Levels of P-selectin. None of the markers show significant difference after conversion to belatacept. All values in ngimL. Error bars signify mean + if- SEM.

Figure 1A shows measurements for Ang-1 and Ang-2. The mean level of Ang-1 at baseline was 7.4 ng/mL (7.9 ng/mL for CNI, 6.9 ng/mL for belatacept). After 12 months after randomization, it was 7.9 ng/mL for CNI again, while it had increased to 8.2 ng/mL in belatacept (*p*-value 0.18). For Ang-2, the average was 2.7 ng/mL (in both groups). Twelve months later it remained at 2.9 ng/mL for both groups. Because Ang-1 and Ang-2 have opposing endothelial effects, the ratio of Ang-2 over Ang-1 is often used to indicate whether a change in concentration has a net activating (rise) or stabilizing (decline) effect on endothelial cells. In CNI-patients, the ratio of Ang-2 over Ang-1 (Ang-2/1 ratio) increased from 0.29 to 0.31 (*p*-value 0.63), while in belatacept it decreased from 0.32 to 0.29 (*p* 0.38) The *p*-value for the difference in Ang-2/1 ratio between CNI and belatacept was non-significant (Mann–Whitney-test, *p* = 0.10).

In Figure 1B, the values for s-TM are depicted. Average s-TM-concentration was 9.2 ng/mL at baseline and decreased to 8.7 ng/mL after 12 months in CNI-patients. For belatacept, the average concentration was 9.3 ng/mL, unchanged. For P-sel (Figure 1C), the average concentration decreased from 32.0 to 29.4 ng/mL in CNI, and was stable in belatacept-patients (27.6 ng/mL to 27.9 ng/mL). When comparing the average change between groups, no difference for either s-TM or P-sel was observed after conversion.

### MiRNAs

**Figure 2:**
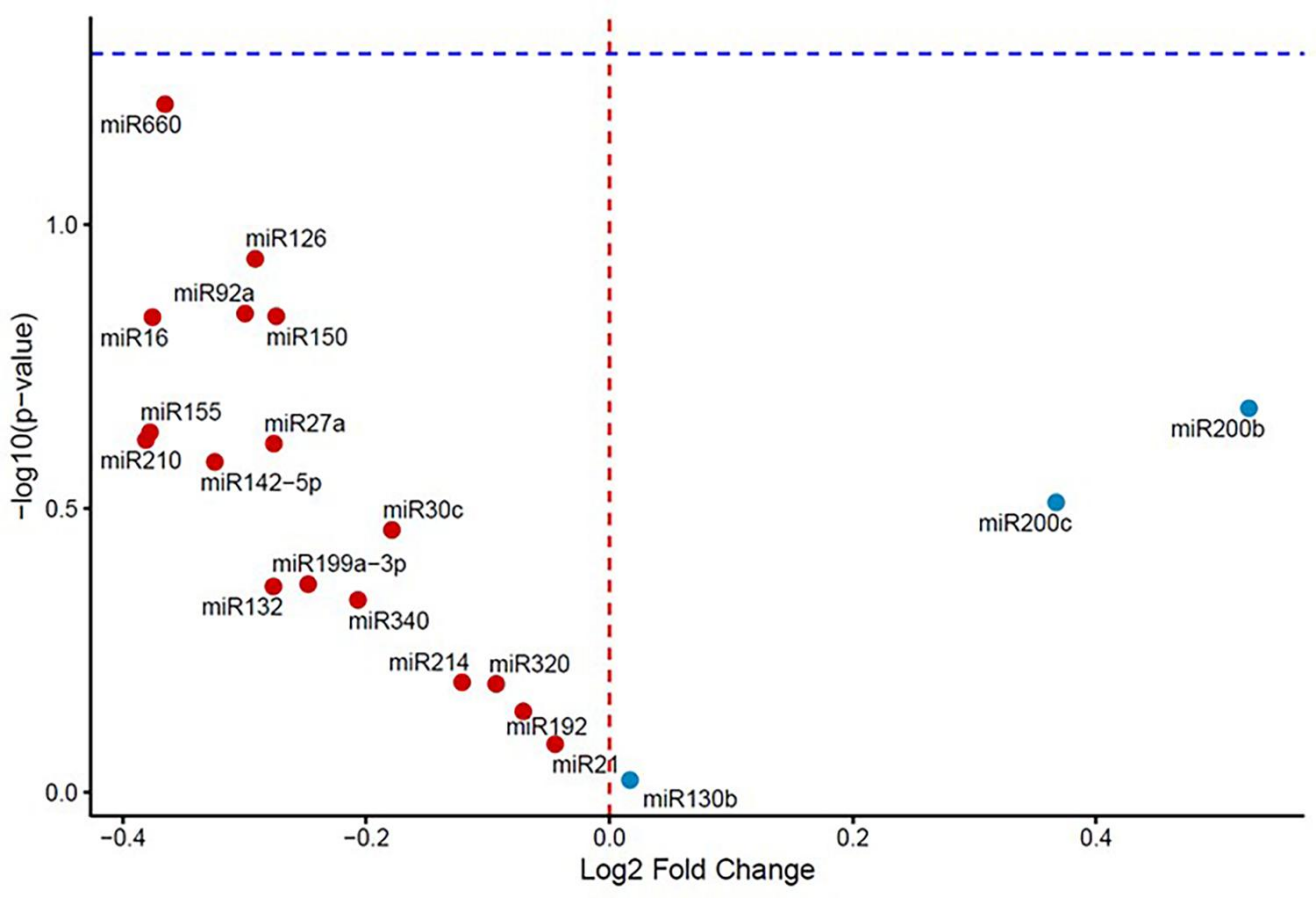
Volcano-plot, showing micro-RNA-fold-change after conversion. The blue line indicates significance (p-value corrected for multiple testing < 0.05). Blue dots represents micro-RNA’s that rise after conversion to belatacept. Red dots represents a decrease in expression. MiR = micro-RNA.

To test if the conversion from tacrolimus to belatacept exerted relevant change on the expression of miRNAs specifically selected for their discriminative properties regarding ED and association with (transplant)-renal function, we performed effect-size analysis of the fold change (FC) of miRNAs. The results are visualized in a Volcano plot (Figure 2). The upper striped blue line represents a *p*-value of 0.05 after correction for multiple testing. It becomes clear from the picture that most miRNAs are expressed in lower quantities after conversion to belatacept than when patients continued using CNI. Only three miRNAs (miR-130b, miR-200c, and miR-200b) increase after conversion while the rest is expressed in lower quantities. However, the changes are small, and none of them are significant.

To visualize whether any clustering-patterns exist between inter-related miRNAs, we performed principal component (PC) analysis (PCA). This methods transforms multiple variables (the miRNAs) into PC's, thereby reducing complexity and enabling the discovery of associated variables. In Supplementary Figure S1, the result of the PCA is shown. No apparent clustering of miRNAs is seen. Also, it confirms the lack of significant change after conversion, indicated by the larger red (CNI) and blue (belatacept) centroid-dots in the middle of the ellipses indicating the averages of all miRNAs, as well as by the low percentage of total variance explained by the main PC's 1 (42.4%) and 2 (11.7%).

### Cell-painting assay: the impact of ciclosporin A and tacrolimus on endothelial phenotype *in vitro*

**Figure 3:**
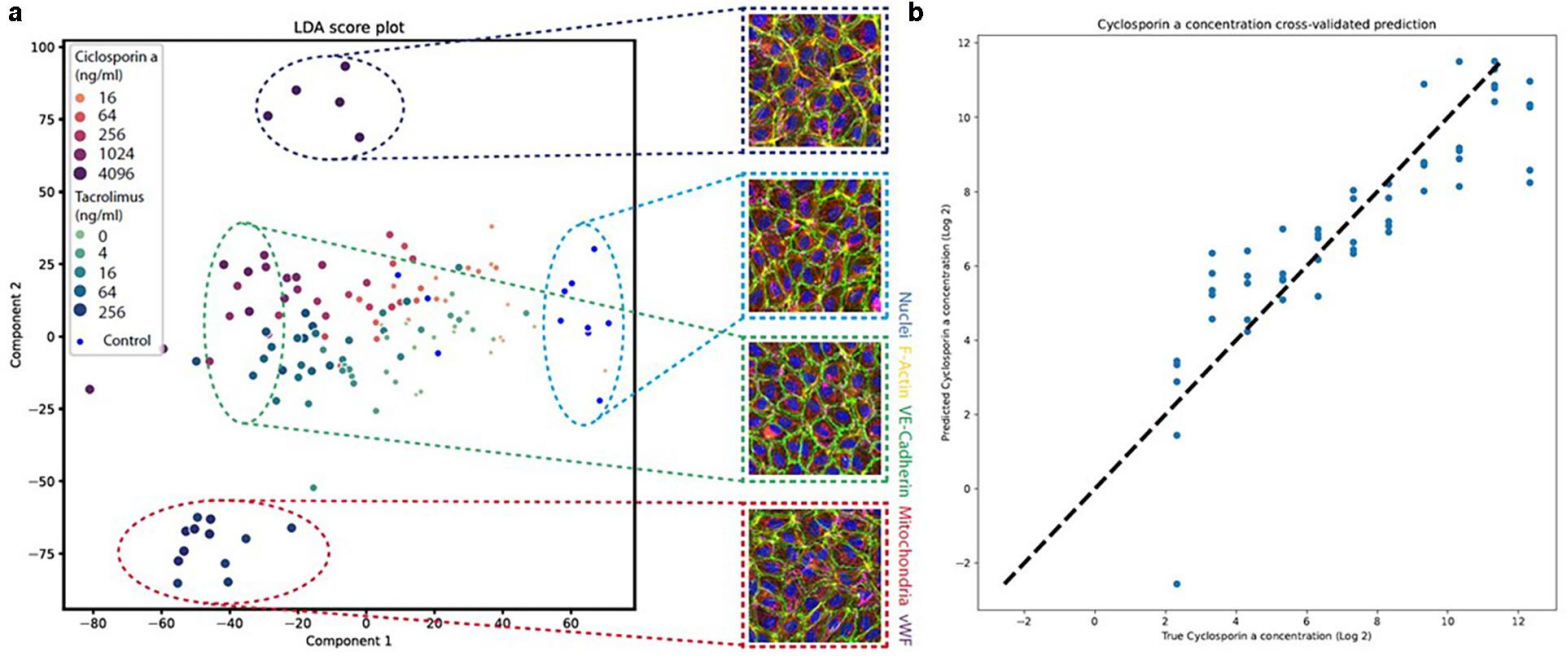
**(a)** linear discriminant analysis of cell painting essays under different concentrations of CNI. Clear distinction of controls (light-blue oval) from high-dose (HD) CsA (dark-purple oval) and HD TA C (dark-blue oval). Green oval contains intermediate doses of both TA C and CsA. **(b)** Regression-fit-plot for estimated vs. true CsA-concentration.

We used linear discriminant analysis (LDA) to assess whether we were able to find a difference in the morphological response of endothelial monolayers on exposure to dose responses of CNI and TAC. It is essential to understand that LDA is a supervised analysis technique, where-as PCA is unsupervised. This means that in LDA-plots, the analysis uses knowledge of group levels [e.g., what CNI-experiment (TAC or CsA), what concentration] to find the combination of variables that best separates the groups. The larger the distance between two samples, the greater the accumulated difference in variables. Figure 3A shows the LDA-plot. On the right side of the picture, images from the experiment are shown. In the actual plot, blue dots represent the control-samples (no CNI). Those samples show endothelial cells in an quiescent state. The cell membranes (green) are broad, and have an intense fluorescence pattern. Stress fibers (yellow) are sparsely seen, and mitochondria (red) are interconnected and located perinuclearly.

The middle of the plot shows a large cloud of samples representing different dilution samples in which higher concentration is visualized by larger dots. A clear gradual change is seen along the horizontal axis, where samples seem to follow a dose-related shift to the left side of the picture. Furthermore, the highest concentrations of both TAC and CsA are represented on the extreme lower and higher margin of the plot respectively. Both groups are clearly discernible from controls and samples. In the images from those patients, we clearly see changes in the cell membrane (less broad, loss of reticular “fishnet” pattern). Besides, the mitochondria show fragmentation, where the interconnection is lost, and the mitochondria are less closely located to the nuclei. Stress fibers (F-actin) are seen more abundantly. In comparison to each other, the intensity of F-actin staining is more pronounced in the higher CsA-concentrations relative to TAC. All the mentioned changes are indicative of an activated state of endothelial cells.

Next, we performed regression-fit-plotting to estimate CNI-concentration based on endothelial morphology. Predicted concentrations were derived from analyzing endothelial cell features using leave-one-out cross validation. The model performed reasonably well for CsA (R^2^ = 0.72, MAE = 1.19 +/- 0.45). In Figure 3B, the results of regression-fit-plotting for CsA are shown. For TAC, we performed the same analysis, which underperformed compared to CsA (Supplementary Figure S2).

### Patient samples

#### Cell-painting assay reveals endothelial changes not related to treatment

**Figure 4:**
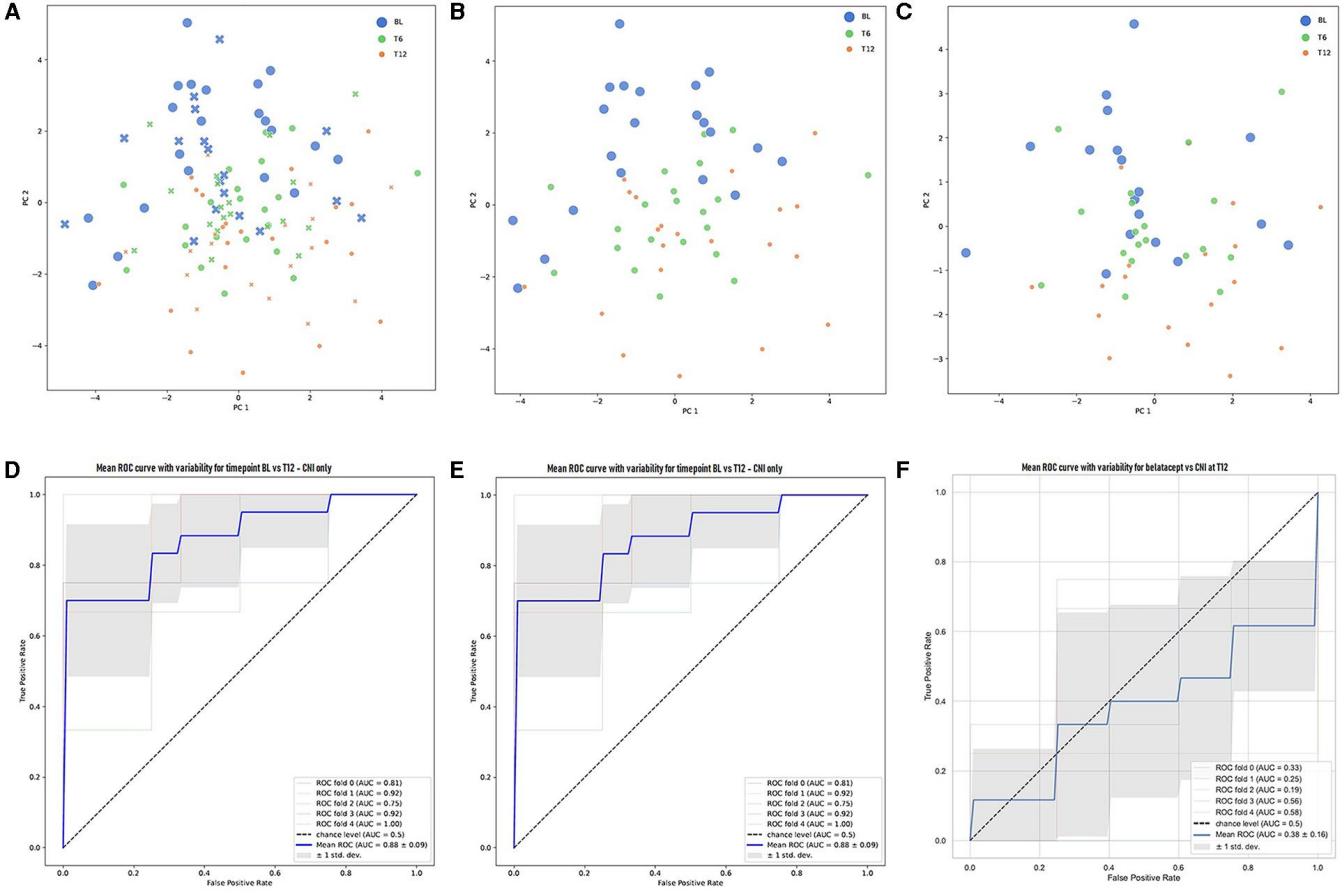
**(d)** ROC curves for cell-painting essays separate BL from T12 for belatacept. **(e)** Same for CNI. **(f)** ROC curves cannot distinguish endothelial cell morphology of belatacept vs. CNI at month 12.

For the patient samples, we also used PCA to visualize patterns in the variables extracted from the cell-images. The results are portrayed in Figure 4A. It shows all the samples, 4b and 4c show the groups. In all patients, we see a migration of the cluster of samples in time. Note that, in contrast to LDA, PCA is non-supervised, meaning it is blind to the group to which the patient-sample belongs. Despite this unsupervised approach, clustering of patients at baseline, at T6 and at T12 is clearly visible, both for belatacept-patients as for CNI (Figures 4b,c). In belatacept-patients, the separation between baseline (when CNI was still used by all patients) and T6 is more pronounced than between T6 and T12. In CNI-patients, the separation is maximal between T6 and T12.

We assembled receiver-operator curves (ROC) to evaluate if samples could be reliably classified to belatacept or control and to what time-point, on the basis of endothelial cell morphology (Figures 4d–f). ROC's correctly classify baseline-patients from T12 patients for both belatacept and CNI. However, they do not reliably differentiate belatacept-patients from CNI-patients at month 12 (Figure 4F). This indicates that the distinction in endothelial cell morphology patterns that is recognized is associated with the factor ‘time after randomization, and not with the result of the randomization itself.

To provide more information on what features are important to separate the samples, we used unsupervised multivariate analysis (factor analysis, FA). Supplementary Figure 3 shows a ranking of the factors identified, and the importance of their contribution to the separation of samples. In factor analysis, the factors have no inherent meaning, but they are constructed from combinations of measured variables that may or may not carry relevance. In our analysis, fifteen factors are identified, of which factor 6, containing information on vWF and mitochondria. Since none of the factors were significantly associated to the conversion in drug-regimen we did not further explore this finding.

## Discussion

In this study, we investigated the effects of a conversion from CNI to belatacept on ED in KTR's. To our knowledge, this study is the first attempt at comparing features of ED in KTR's randomized to belatacept. We believe that the use of samples from patients in international RCT's is a major strength of our approach. Also, the use of low-dose TAC in 90% of our patients is similar to current international practice, which helps external validation of the results. On the other hand, the predominance of TAC means that we must be careful in generalizing our findings to CNIs as a drug class. The loss of samples, as described in the results section is an obvious limitation. Another shortcoming is that all our markers of ED are taken from blood samples. Although the study protocol of the SMR-2729 enabled us to evaluate histologic results, biopsies were only taken in a very limited number of cases, all concerning the clinical suspicion of rejection. Therefore, we did not include an evaluation of local (kidney graft) endothelial damage, like arterial hyalinosis, or inflammatory changes to the microvascular department. Neither did we collect information on proteinuria, which is also a feature of microvascular damage to the graft. Lastly, although we believe to be the first to report on ED after CNI-conversion, a word of caution is warranted as most patients in our study were included from the only “negative” (conversion from CNI to belatacept did not improve GFR) conversion-study published, beside a very small trial by de Graav ea, that showed extreme rates of rejection in the first year (16, 42). Our most striking conclusion is that we found no significant change in ED in the biomarkers we explored. Neither traditional biomarkers of vascular function, nor miRNAs, nor our novel “cell-painting” assay showed any marked change in patients on belatacept when compared to patients that continued to use CNI.

The angiopoietin system involves two antagonizing components: Ang-1 is secreted by pericytes underlying the endothelium, binding to it through its Tie-2-receptor (Tie-2R). Ang-2 is released from Weibel-Palade bodies in conditions like sepsis. Ang-1 promotes vessel integrity, while Ang-2 expels Ang-1 from Tie-2R, stimulating detachment of endothelial cells from pericytes and promoting vascular leakage. We had expected our study to show a decrease in Ang-2/1 ratio, indicative of a shift towards healthy, stabile endothelial structure. In transplantation, early skewing of the angiopoietin system towards Ang-2 after ischemia-reperfusion injury (IR/I) is a predictor of pericyte loss and capillary rarefaction (43, 44). Previous work from our own lab proved Ang-2/1 ratio changes one year after simultaneous pancreas-after-kidney transplantation (SPK) and kidney-rejection (23, 45).Animal studies have associated TAC with suppression of Ang-1 *in vitro*, while Ang-2-upregulation is also implied in TAC-associated hypertension (21, 46). Based on our observations, we had expected our current study to show a decrease in Ang-2/1 ratio after a year, reflecting improved vascular health after conversion to belatacept. However, we found no change in Ang-1, nor Ang-2, and just a non-significant decrease in Ang-2/1 ratio.

We also investigated the effect of conversion on s-TM and P-sel. S-TM is shed from endothelial cells after inflammatory injury. Previous work from our lab identified s-TM to be increased in kidney failure. S-TM was rapidly reversed after transplantation and restoration of renal function, only to rise again in those patients developing interstitial fibrosis and tubular atrophy (47). Since IF/TA is one of the features of CNI-toxicity in renal transplant-biopsies we had hypothesized that s-TM would possibly be decreased in patients weaned of CNI. However, we did not see such difference. The lack of difference was also found for P-sel, a thrombocyte-derived marker of endothelial activation (48) and ED, and the third traditional biomarker studied (23, 43, 49–51). Besides the obvious conclusion that belatacept does nothing to change the concentration of these agents, it is also possible that any effect that would have occurred was missed due to suboptimal timing of sampling, as changes in concentrations are thought to be dynamic (23, 45). However, the ratio of Ang-2/1 remained elevated in rejection patients inthe previous experiment in our lab mentioned above (23). Of note, in that study, the mean eGFR in those that had rejection was 11 mL/m/1.73m^2^ lower than in those with stable GFR. The changes in Ang 1/1-ratio and s-TM in that study remained statistically significant after correction for loss of GFR. As we also observed a difference in eGFR in the baseline features of belatacept patients, we must consider the fact that CKD added to ED more in belatacept-patients than in controls. However, it is probably safer to conclude that conversion from CNI to belatacept is of far more limited effect on ED than having rejection. It is also possible that our follow-up of one year was too short to witness any effect. For instance, the BENEFIT-studies revealed incremental difference in eGFR between belatacept and CsA, even after seven years (52).

The second part of our investigations, on miRNA-expression, revealed no significant changes either. We must emphasize again that we could only analyse samples of 70 patients, instead of 100, as markings of samples were unfortunately lost during storage. It is possible that we would have detected significance in a larger sample size. We had selected 20 miRNAs that were previously associated with ED, kidney fibrosis and restoration of kidney function after kidney transplantation (24–35). We had expected several of those to be differentially expressed after conversion to belatacept. Previous literature, including our own showed excellent biomarker-potential of miRNAs like miR-126 and miR-132 in transplantation-settings of IR/I, rejection and graft dysfunction (23, 27, 33). Others, like miR-21, miR-214 and miR-199a have been suggested to play a role in CNI-related toxicity (31). Again, we did not observe differences to our selected miRNAs. Interestingly, Neal e.a. previously showed the total concentration of miRNAs to be lower in patients with severe CKD. In this regard, we have to consider again that the belatacept-group had lower GFR, and a lower total amount of RNA may have made it harder to detect a change in concentration, despite the fact that most of our patients had renal function in the CKD-3-range, where Neal showed the most relevant differences in CKD 4 and 5 (35).

We saw that almost all miRNAs except three were downregulated after belatacept-conversion. Although the lower expression itself did not reach significance from any individual miRNA, we must consider some explanations for overall-lower expression of genetic material. One is subclinical viral infection, as some viral infections are known to interfere with mRNA-transcription through inhibition and degradation of RNA-polymerase II (53, 54). We have previously shown Torque Teno Virus (TTV) concentrations to be higher in belatacept-treated patients (55). Secondly, since miRNAs are shed from vesicles that originate from circulating cells, lower numbers of circulating cells may also be implicated. We have previously shown that conversion to belatacept is associated with lower numbers of T-cells, B-cells and some subclasses of monocytes (56). Finally, the use of RNU44, RNU48, and U6 as reference controls for circulating miRNA analysis may not be optimal, as these are primarily cellular small RNAs and may not perfectly reflect RNA amounts in circulating biofluids.

In our dilution-series cell-painting experiments, we showed dose-dependent effects of CNI on endothelial cell morphology. Gradual loss of VE-cadherin-staining and the appearance of F-actin stress fibers under influence of CsA have been shown before in cultured pulmonary endothelial cells, albeit in concentrations 200-20,000 times the highest concentration in our study (57). Effects on EC-mitochondria have also been described, but are somehow two-pronged. Calcineurin is essential for the translocation of dynamin-related protein-1 (DRP1) to mitochondria and its activation by dephosphorylation. When activated, DRP-1 causes mitochondrial fragmentation and ultimately cell death. Both CsA and TAC revert this process, protecting against mitochondrial dysfunction (58). On the other hand, calcineurin inhibition causes mitochondrial accumulation of calcium, and the ensuing result is the production of toxic reactive oxygen species (59, 60). In the literature, more examples of this protective effect of CNI can be found. For instance, CNI have an attenuating effect on ED after brain death in a pig model, and earlier studies have shown protection against IR/I-induced ED in multiple animal models (although clinical studies did not corroborate the results in humans) (61–64).

In our last experiment, we used the cell painting model to test the effect of patient plasma samples on endothelial cell morphology. As such, we could compare the differential effects of belatacept, - and CNI-treatment, incorporating the effects of comorbidities such as age, diabetes and CKD. Although we did see endothelial morphology change after six and twelve months, we did not see a treatment effect, as the cell changes were observed in both groups, and the belatacept-samples did not show reversal of toxicity. Of note, the dose-response curve of CNI with endothelial changes seems to be stronger for CsA than for TAC in the dilution-series experiment (Figure 3b). Most patients (over 90%) represented in the samples used TAC, in “low” (trough-level 3–7) doses. Not finding a treatment effect might reflect the fact that such a dose is below the toxic threshold for endothelial (renal) cells. So, although our results suggest a clear toxic effect of increasing doses of both CsA and TAC on endothelial cell structure *in vitro*, it is clear that this model of ED was not significantly influenced by the presence of serum derived from patients on belatacept or CNI, indicating that clinically-relevant ED depends on other comorbidities, or that ED due to TAC is largely dependent on dosis. In that regard, our observations fit in a longer-standing debate that challenges the importance of “chronic CNI-toxicity”. For instance, Issa ea argue that, although there is no doubt about the existence of acute CNI-related nephrotoxicity due to (reversible) vasoconstriction, the attribution of loss of GFR and CKD to CNI-use is questionable (65). The histologic changes that are seen in chronic CNI-use can also be seen in chronic rejection due to anti-HLA-antibodies (ABMR) (66) and are thus non-specific.

In conclusion, we have not been able to show significant changes in ED after conversion from CNI (mostly TAC) to belatacept. no steroids were used. Beside the possibility that the agents we investigated were subject to dynamic changes in concentration, and that our one-time sampling after a year was insufficient to detect any change, it seems reasonable to conclude that other vaso-toxic influences like CKD, diabetes, age, comorbidities and the use of steroids over-ride the impact of current low-dose CNIs on ED, assuming there still is a clinical effect.

In conclusion, we have investigated whether a conversion of immunosuppressive regimen resulted in early changes in features of ED in KTR, and saw surprisingly little difference between patients on CNI and those converted to belatacept. Although several limitations in interpreting the results of our investigation are discussed, the hypothesis that CNI-related toxicity in a clinical setting is due to endothelial dysfunction, or that ED can be mitigated by conversion to belatacept is not supported in our study.

## Data Availability

The raw data supporting the conclusions of this article will be made available by the authors, without undue reservation.
