# Supplementary material for: An exploration of markers of microvascular dysfunction in kidney transplant recipients randomized to belatacept: no clinical impact of CNIs on endothelial function
Source: Front Transplant. 2026 Jun 11;5:1812847. doi: 10.3389/frtra.2026.1812847 (PMC13294043; doi:10.3389/frtra.2026.1812847)
Supplement: Supplementary file 2 [file Presentation1.pptx]

## Slide 1
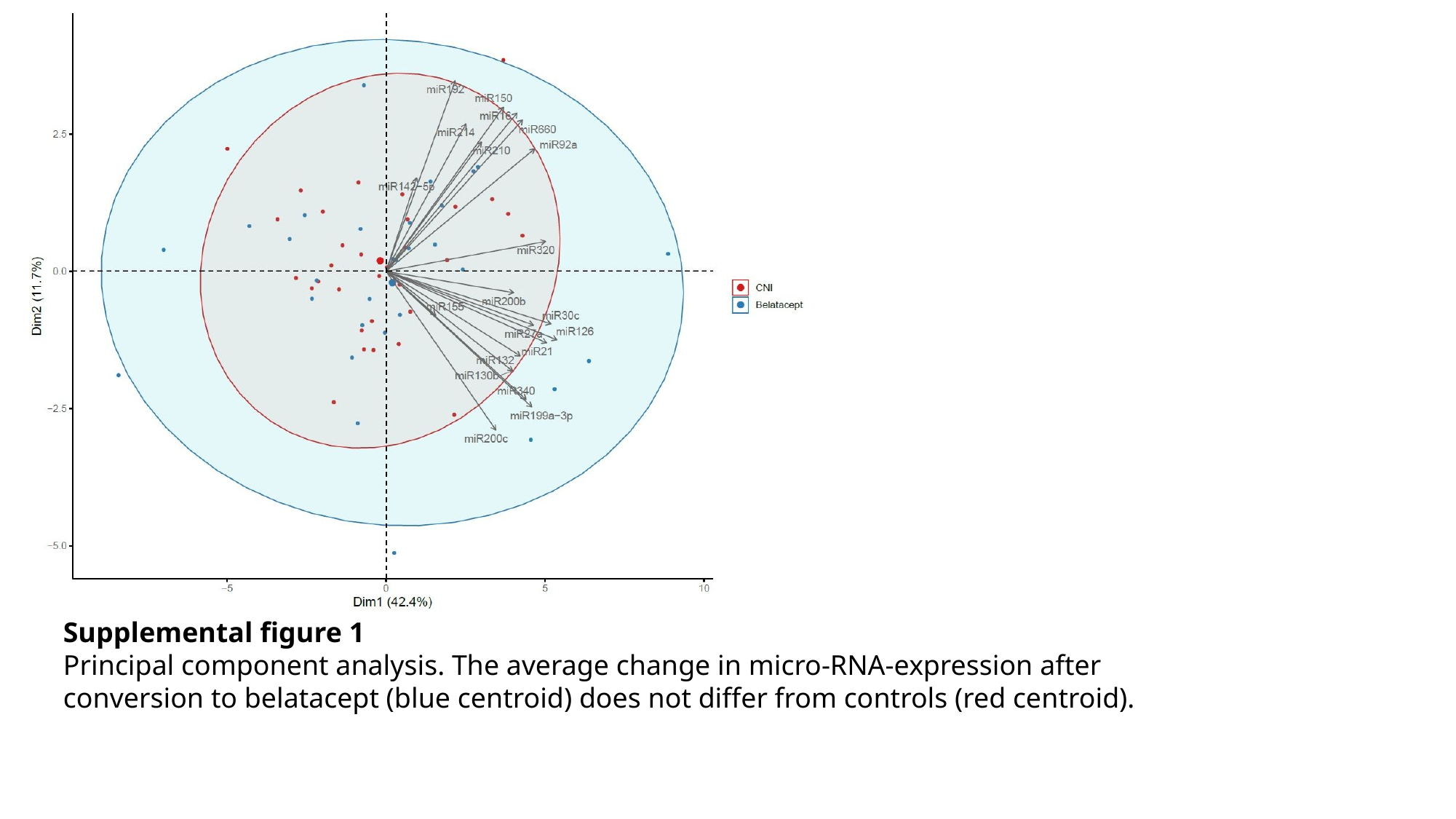

Supplemental figure 1
Principal component analysis. The average change in micro-RNA-expression after conversion to belatacept (blue centroid) does not differ from controls (red centroid).
