# Supplementary figures and images for: An exploration of markers of microvascular dysfunction in kidney transplant recipients randomized to belatacept: no clinical impact of CNIs on endothelial function
Source: Front Transplant. 2026 Jun 11;5:1812847. doi: 10.3389/frtra.2026.1812847 (PMC13294043; doi:10.3389/frtra.2026.1812847)

## Slide 1
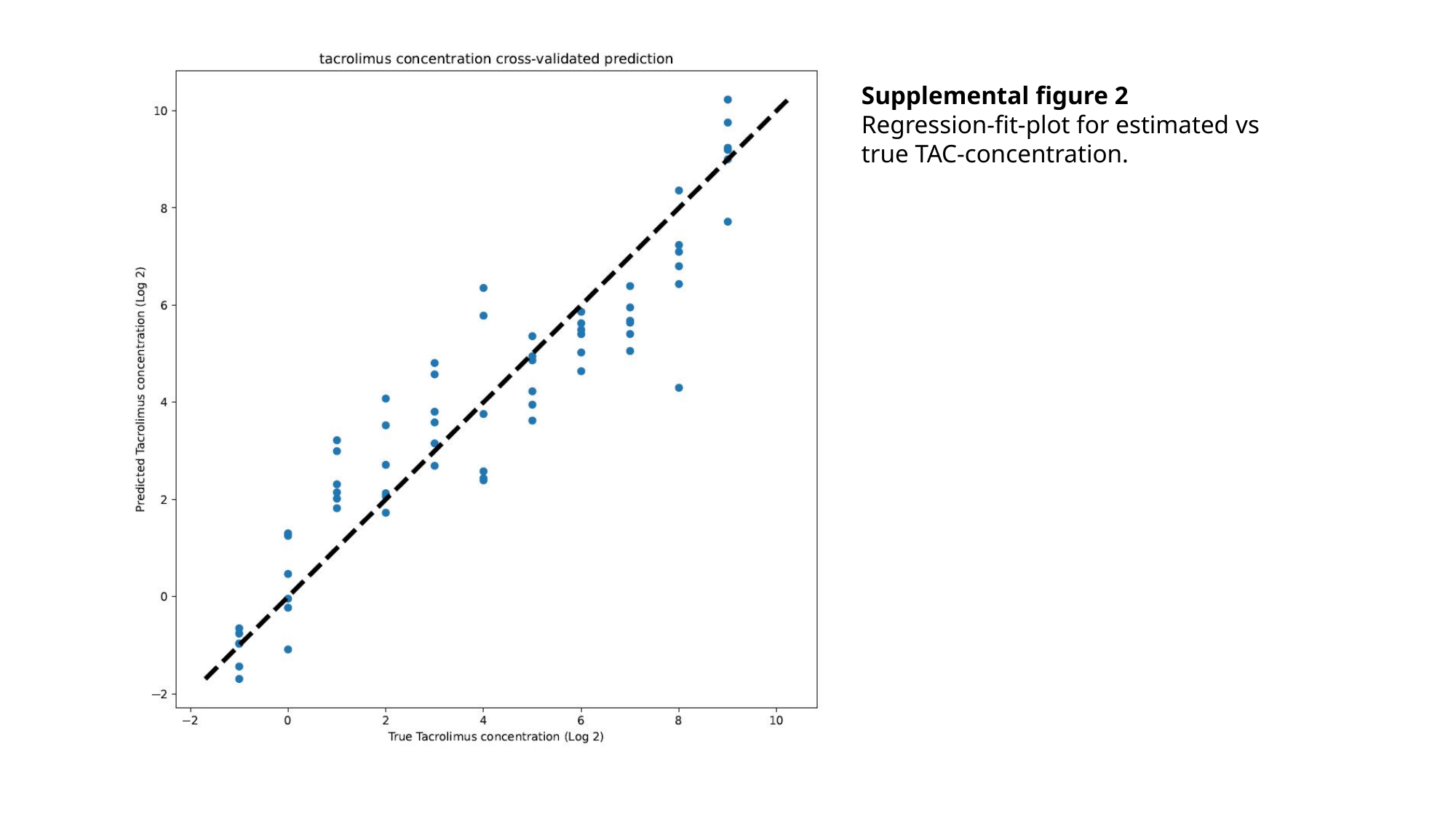

Supplemental figure 2
Regression-fit-plot for estimated vs
true TAC-concentration.

Supplement: Supplementary file 3 [file Presentation2.pptx]
