# Supplementary material for: An exploration of markers of microvascular dysfunction in kidney transplant recipients randomized to belatacept: no clinical impact of CNIs on endothelial function
Source: Front Transplant. 2026 Jun 11;5:1812847. doi: 10.3389/frtra.2026.1812847 (PMC13294043; doi:10.3389/frtra.2026.1812847)
Supplement: Supplementary file 4 [file Presentation3.pptx]

## Slide 1
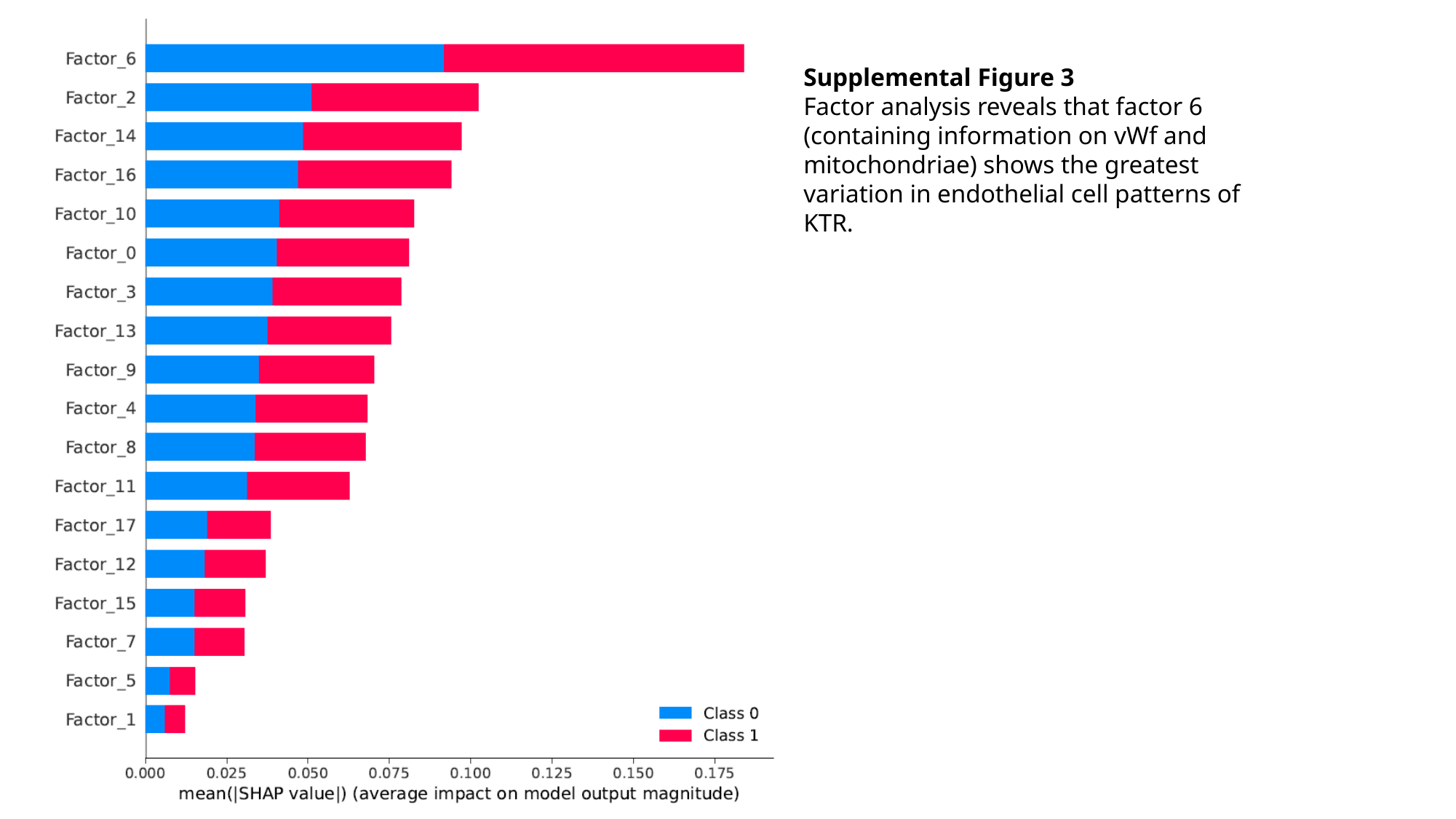

Supplemental Figure 3
Factor analysis reveals that factor 6 (containing information on vWf and mitochondriae) shows the greatest variation in endothelial cell patterns of KTR.
