# Supplementary material for: An exploration of markers of microvascular dysfunction in kidney transplant recipients randomized to belatacept: no clinical impact of CNIs on endothelial function
Source: Front Transplant. 2026 Jun 11;5:1812847. doi: 10.3389/frtra.2026.1812847 (PMC13294043; doi:10.3389/frtra.2026.1812847)
Supplement: Supplementary file 5 [file Presentation4.pptx]

## Slide 1
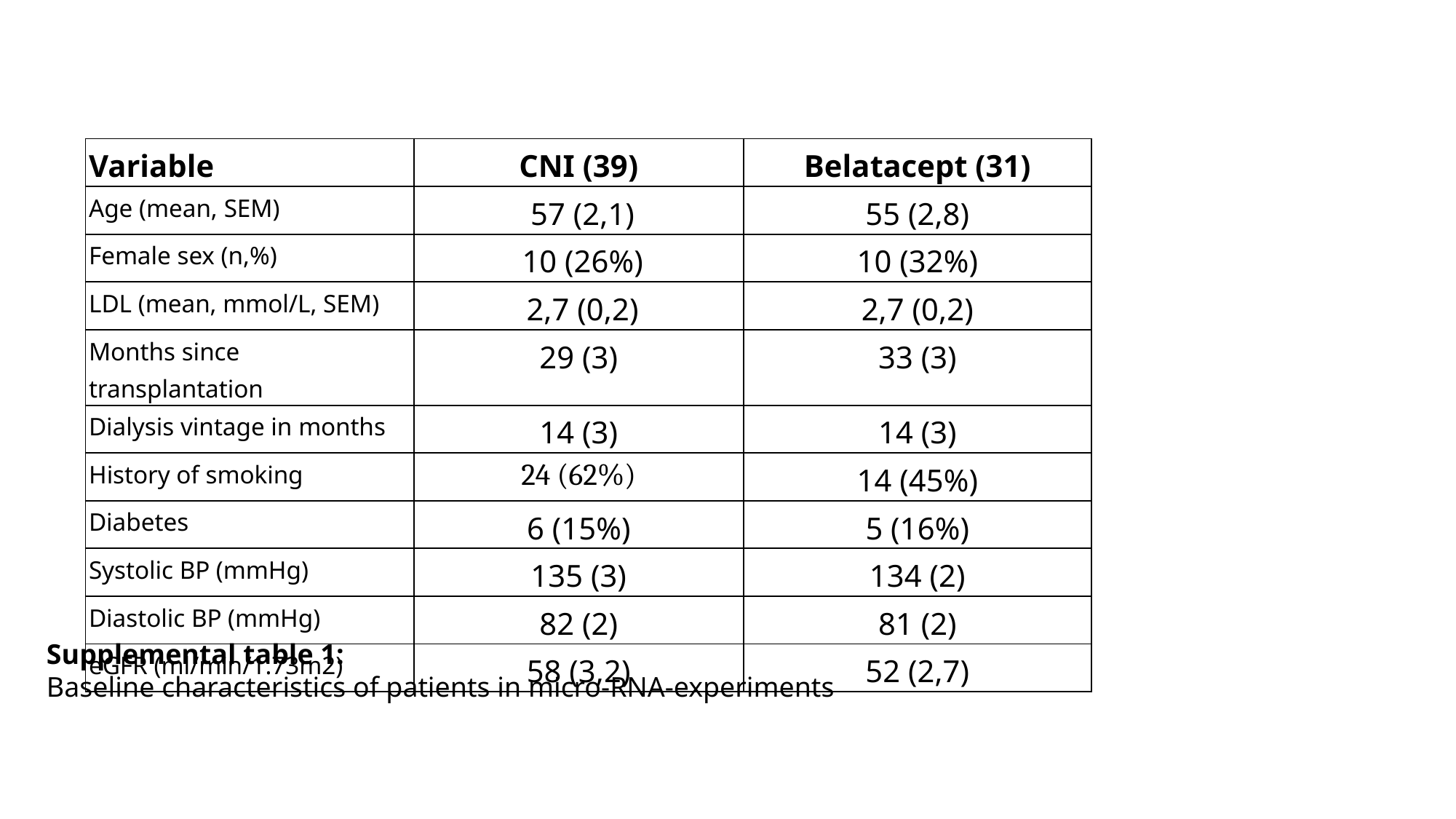

| Variable | CNI (39) | Belatacept (31) |
| --- | --- | --- |
| Age (mean, SEM) | 57 (2,1) | 55 (2,8) |
| Female sex (n,%) | 10 (26%) | 10 (32%) |
| LDL (mean, mmol/L, SEM) | 2,7 (0,2) | 2,7 (0,2) |
| Months since transplantation | 29 (3) | 33 (3) |
| Dialysis vintage in months | 14 (3) | 14 (3) |
| History of smoking | 24 (62%) | 14 (45%) |
| Diabetes | 6 (15%) | 5 (16%) |
| Systolic BP (mmHg) | 135 (3) | 134 (2) |
| Diastolic BP (mmHg) | 82 (2) | 81 (2) |
| eGFR (ml/min/1.73m2) | 58 (3,2) | 52 (2,7) |
Supplemental table 1:
Baseline characteristics of patients in micro-RNA-experiments
