# Supplementary material for: An exploration of markers of microvascular dysfunction in kidney transplant recipients randomized to belatacept: no clinical impact of CNIs on endothelial function
Source: Front Transplant. 2026 Jun 11;5:1812847. doi: 10.3389/frtra.2026.1812847 (PMC13294043; doi:10.3389/frtra.2026.1812847)
Supplement: Supplementary file 6 [file Presentation5.pptx]

## Slide 1
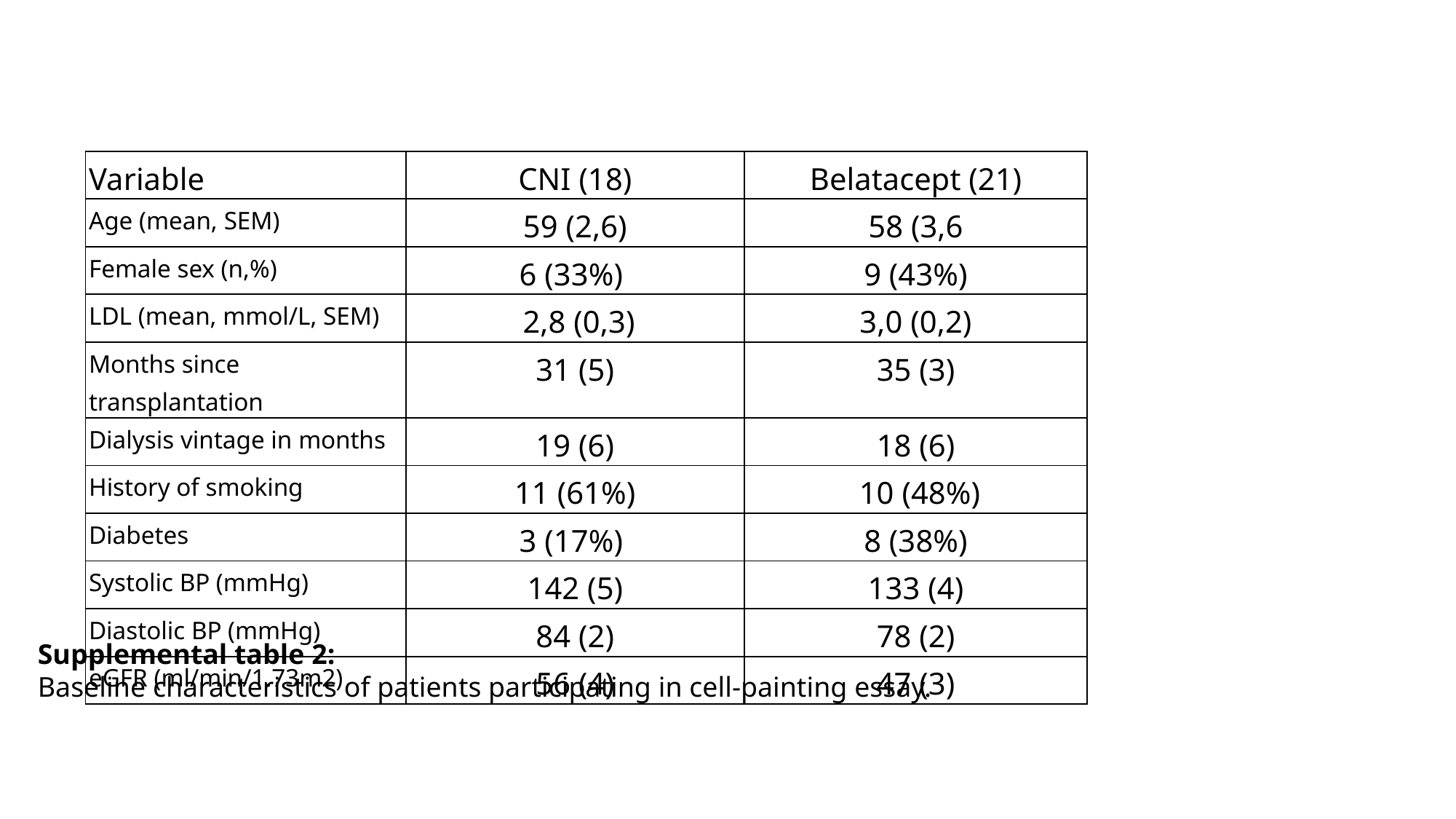

| Variable | CNI (18) | Belatacept (21) |
| --- | --- | --- |
| Age (mean, SEM) | 59 (2,6) | 58 (3,6 |
| Female sex (n,%) | 6 (33%) | 9 (43%) |
| LDL (mean, mmol/L, SEM) | 2,8 (0,3) | 3,0 (0,2) |
| Months since transplantation | 31 (5) | 35 (3) |
| Dialysis vintage in months | 19 (6) | 18 (6) |
| History of smoking | 11 (61%) | 10 (48%) |
| Diabetes | 3 (17%) | 8 (38%) |
| Systolic BP (mmHg) | 142 (5) | 133 (4) |
| Diastolic BP (mmHg) | 84 (2) | 78 (2) |
| eGFR (ml/min/1.73m2) | 56 (4) | 47 (3) |
Supplemental table 2:
Baseline characteristics of patients participating in cell-painting essay.
