# Supplementary material for: An exploration of markers of microvascular dysfunction in kidney transplant recipients randomized to belatacept: no clinical impact of CNIs on endothelial function
Source: Front Transplant. 2026 Jun 11;5:1812847. doi: 10.3389/frtra.2026.1812847 (PMC13294043; doi:10.3389/frtra.2026.1812847)
Supplement: Supplementary file 1 [file Table1.docx]

Leiden, 04-03-2026

Dear editor,

On behalf of the co-authors I want to thank the editing team for the chance to present our research to you. I want to highlight one chance I made to the manuscript after the review of the reviewers. In the second-to-last paragraph of the discussion section, I re-wrote a sentence. The last sentence of that paragraph now reads:

‘and the use of steroids over-ride the impact of current low-dose CNIs on ED, assuming there still is a clinical effect’

instead of

‘and the use of steroids over-ride the impact of current low-dose CNI’s impact on ED’.

Furthermore, I added ‘figure 1c’ and removed the ‘a’ from ‘figure 2a’ in the text where there is a reference to those figures. Unfortunately, I had not changed that in time for the reviewers to be able to see that. I assume it does not necessitate a new round of revision, but in view of transparency, I hereby want to make you aware of that discrepancy.

Kind regards,

Edwin Bredewold

First author
